# Impact of an open healing approach on peri-implant mucosa following immediate implant placement with transmucosal provisionalization: a systematic review and meta-analysis

**DOI:** 10.1186/s12903-026-08105-z

**Published:** 2026-03-20

**Authors:** Charles C. Zadikian, Melodie M. Clerc, Jean-Louis Zadikian, Stéphane Kerner, Lucas Duong, Juliane Isaac, Benjamin P.J Fournier

**Affiliations:** 1 Faculty of Dental Surgery, Université Paris Cité, Paris, France; 2 Université Paris Cité and Sorbonne Paris Nord, Inserm, Santé Orale, Montrouge, F-92120 France; 3 Reference Center for Oral and Dental Rare Diseases ORARES, APHP, Rothschild Hospital, 5 rue Santerre, Paris, 75012 France; 4 Dental Department, Charles Foix Hospital, APHP, Ivry-Sur-Seine, France; 5 Department of Periodontics, School of Dentistry, Loma Linda University, Loma Linda, CA USA; 6 Post-Graduate Program in Periodontology and Implant Dentistry, Université Paris Cité, EFP, Paris, France; 7 French Association of Implantology for General Dental Practitioners, AFOPI, Sarcelles, France

**Keywords:** Immediate dental implant placement, Open-healing, Bone grafting, Peri-implant mucosa, Immediate provisionalization, Systematic review, Meta-analysis

## Abstract

**Objective:**

To compare the peri-implant mucosal stability between an “open healing” approach (test) during immediate dental implant placement with bone grafting and immediate transmucosal provisionalization (non-occlusal) versus primary flap closure (control).

**Methods:**

This systematic review followed the PRISMA guidelines. A literature search was conducted in multiple databases up to May 2025. The primary outcome was peri-implant mucosal stability (Midfacial Mucosal Level (MML). Secondary outcomes include peri-implant soft tissue parameters (e.g. Papilla evaluation, mucosal thickness) and bone related outcomes (e.g. Marginal Bone Loss (MBL), buccal bone thickness and implant survival rate).

**Results:**

Out of 362 records, 12 studies were included, representing 431 patients and 438 implants. Most studies demonstrated a low risk of bias, although heterogeneity in outcomes assessment was observed. Meta-analysis showed limited midfacial mucosal changes between control and test groups, showing good stability of peri-implant keratinized tissue. Other soft and hard tissue outcomes were comparable between groups.

**Conclusion:**

The open healing approach with bone particle grafting during immediate dental implant placement appears as a viable option for supporting midfacial mucosa and maintaining mucosa and bone stability. However, more high-quality randomized controlled trials are necessary to confirm these findings.

**Clinical Relevance:**

This minimally invasive protocol appears to provide satisfactory outcomes by preserving peri-implant tissues, thereby reducing the need for multiple surgical interventions.

**Supplementary Information:**

The online version contains supplementary material available at 10.1186/s12903-026-08105-z.

## Introduction

In clinical scenarios necessitating the replacement of a compromised tooth, protocols involving single immediate dental implant placement coupled with immediate non-loaded provisionalization have demonstrated high cumulative survival rates [1–3]. This includes placing a dental implant directly after the tooth extraction into the fresh extraction socket. Then a non-functional provisional restoration is screwed on the same day. This method minimizes surgical interventions and offers an immediate fixed aesthetic solution to patients [4].

Post-extraction dimensional changes in the alveolar socket could be observed, particularly within the first six months post-extraction, characterized by notable bone resorption. Facial bone wall thickness is a critical factor influencing bone resorption. Thin bone wall phenotypes (1 mm or less) exhibit progressive bone resorption with a vertical loss, whereas thick bone wall phenotypes undergo only minor bone resorption. In addition, thin bone wall phenotypes often demonstrate a spontaneous soft tissue thickening, while thick bone wall phenotypes show no significant changes in soft tissue dimensions after 8 weeks of healing [5].These findings underscore the dynamic changes in both hard and soft tissues post-extraction, particularly in patients with thin bone phenotypes, highlighting the need for strategies like “open-healing” to preserve the alveolar ridge and optimize peri-implant mucosa stability.

Grafting bone materials into the gap, between the implant surface and the socket walls immediately after implant placement, helps to reduce the alveolar ridge resorption [6]. Although autografts remain the gold standard, a variety of grafting materials, including allografts, xenografts, and synthetic options, are also used to fill this gap, with their integration and healing properties in bone tissue being well-documented [7].

In the field of alveolar ridge preservation, several techniques are employed to maintain the integrity of the tooth socket post-extraction. One such technique is the “open-healing” approach, which does not require the primary closure of the socket by covering the bone graft with a collagen matrix. Instead, an alternative to this method utilizes a provisional restoration, or alternatively a customized healing abutment, as a protective seal over the socket to stabilize the bone graft, allowing for a secondary intention healing processes, without a barrier membrane [8]. This method offers significant advantages, reducing surgical trauma and eliminating the need for invasive procedures such as a coronally advanced flap or a free gingival graft [9]. It aims to minimize surgical intervention while promoting the retention of alveolar ridge dimensions and the health of the mucogingival junction. Studies have demonstrated its effectiveness in preserving vital bone [10, 11].

Other factors, including anatomical features or prosthetic design, play crucial roles in the remodeling of peri-implant hard and soft tissues. Among these, supracrestal tissue height has been identified as a key determinant [12, 13] : encompassing the sulcular epithelium, junctional epithelium, and supracrestal connective tissue, from the mucosal margin to the crestal bone [14], and its phenotype is essential for the maintenance and protection of peri-implant tissues [15]. A deeper understanding of how particle bone grafting materials interact with this zone under “open healing” conditions is needed. Indeed, the effects of this method on soft tissue integration and aesthetic outcomes has not been thoroughly explored. Although in vivo studies report bone grafting materials incorporated into supracrestal soft tissues without inflammatory reactions [16], they do not provide conclusions about the clinical outcomes nor oral soft tissue healing process. When the provisional restoration is disconnected during the prosthetic phase, bone substitute particles may be clinically spotted, incorporated into the peri-implant soft tissue emergence profile [17]. Some recent protocols go one step further by also advising the placement of bone grafting materials above the crestal level, into the soft tissue zone to achieve an optimal peri-implant soft tissue architecture [18]. To date, the effects of the incorporation of bone grafting materials into supracrestal soft peri-implant tissues remain unexplored.

This systematic review and meta-analysis focuses on the clinical outcomes of peri-implant mucosa following an “open-healing” approach during immediate dental implant placement with non-occlusal provisionalization associated with a bone grafting, meaning without a barrier membrane. We assess peri-implant mucosal stability, while secondary outcomes include peri-implant soft tissue parameters, bone remodeling outcomes, and implant survival rate. We aim at providing evidence-based recommendations for immediate dental implant placement with the open healing approach.

## Methods 

### Protocol and registration

This study was conducted in accordance with the Preferred Reporting Items for Systematic Reviews and Meta-Analyses statement [19] and registered (CRD42023439073) in the International Prospective Register of Systematic Reviews.

### Search strategy

Literature searches were conducted in English and French language articles in May 2025 by 2 investigators (C.Z and M.C.) in the PubMed (MEDLINE), EMBASE, Dentistry of Oral Science Source, and Cochrane databases. The following MeSH terms were used: (immediate dental implant loading), (immediate implant placement), (immediate loading), (bone regeneration), (bone substitute), (guided bone regeneration), (oral mucosa), (gingiva). The search was adjusted for each database as specified in Supplemental Table 1.

An AI-based language tool was used to improve English clarity. The authors remain fully responsible for the content.

### PICO question Inclusion and exclusion criteria

The research question was:

In healthy patients requiring a tooth extraction (*population)*, what is the effect of an open healing approach during an immediate implant placement with immediate transmucosal provisionalization (non-occlusal) associated with bone grafting (*intervention)* on peri-implant soft tissue *(outcome)*?

The objectives of this systematic review were:

- To assess peri-implant mucosal stability.
- To evaluate peri-implant soft tissue parameters, bone remodeling outcomes, and implant survival rate.

The Population, Intervention, Comparison, and Outcomes (PICO) framework was used:

- *Population*: Adult patients undergoing immediate implant placement.
- *Intervention*: Open-healing technique: immediate transmucosal provisionalization (non-occlusal) with bone grafting.
- *Comparison*: Bone grafting with primary closure.
- *Outcome*: Primary outcome: Midfacial Mucosal Level (MML).
- *Secondary outcomes*: Papilla evaluation, Peri-implant mucosa thickness, Buccal horizontal alteration, Pink Esthetic Score (PES), Keratinized Mucosa Width (KMW), Marginal Bone Loss (MBL) and Buccal Bone Thickness (BBT), Implants survival rate.

#### Definitions used in this article

Immediate implant placement refers to implant insertion at the time of tooth extraction.

Immediate provisionalization refers to the immediate connection (within 1 week) of a transmucosal component (healing abutment or provisional restoration), maintained out of occlusion.

Immediate loading refers to an implant restoration placed in direct occlusal contact within 48 h after placement.

In the context of the present review, the term “open-healing” refers to transmucosal management of the extraction socket using either an anatomic healing abutment or a provisional restoration without primary flap closure.

### Inclusion and non-inclusion criteria

Inclusion criteria:

- Population: Systemically healthy patients ≥ 18 years old with an indication for tooth extraction.
- Intervention: Treatment of a single tooth with an immediate implant with immediate transmucosal provisionalization (non-occlusal) associated with bone grafting using open healing. Variations in design (customized healing abutment or a provisional restoration) were considered as transmucosal provisionalization.
- Comparison: Primary flap closure.
- Outcome: Primary outcome: MML. Secondary outcomes: Papilla evaluation, Peri-implant mucosa thickness, Buccal horizontal alteration, PES, KMW, MBL and BBT, Implants survival rate.
- Study design: Journal article until June 2024: Randomized Clinical Trials (RCTs) of parallel design, as well as prospective case series with at least 5 patients and with a follow-up that includes the placement of final restorations.

Non-inclusion criteria:

- Studies reporting no data on soft tissue.
- Protocols include a gingival graft or a soft tissue substitute.
- Retrospective studies, case reports, review papers, conference abstracts, and opinion articles.

Studies involving immediate functional occlusal loading (defined as provisional restorations placed in direct occlusal contact within 48 h after implant placement) were excluded. Only protocols with non-occlusal immediate provisionalization or transmucosal healing abutments during the early healing phase were considered eligible. In all included articles, none reported provisional restorations in occlusal contact during the early healing phase. Therefore, the present analysis strictly evaluates soft tissue management strategies and not the effect of mechanical functional loading.

Only randomized clinical trials (RCTs) were considered for meta-analysis.

### Data extraction and risk of bias (RoB) assessment

All titles and abstracts of the articles retrieved were independently assessed by two reviewers (C.Z. and M.C.) using the Rayyan online software (Qatar Computing Research Institute) [20]. Rayyan was also used to find and consider duplicate publications. Articles were initially selected based on titles and abstracts. Potentially relevant articles were selected for full text screening. The remaining articles were extracted for independent sorting by the reviewers. In the case of a selection conflict, a third reviewer (B.F.) made the decision. Two reviewers (C.Z and M.C) independently performed quality assessment following the Cochrane Collaboration’s tool for assessing RoB in RCTs (RoB2) [21] and the Joanna Briggs Institute evaluation tool for cohort studies [22], case-control and case series studies. Level of evidence of each article was evaluated using Oxford Centre for Evidence-Based Medicine OCEBM Levels of Evidence [23].

Data from the included articles were extracted into a dedicated Excel spreadsheet. If needed, the authors of the selected studies were contacted for further information.

### Statistical analysis

The Cohen kappa coefficient (κ) was used to compute the agreement rate between the primary and secondary reviewers after full-text analysis. Data were analyzed using (Prism, Version 10.2.3, Graphpad Software, LLC). A meta-analysis was performed if more than one study (RCT) provided homogeneous information regarding a specific outcome. The chi-square test (α = 0.05) and the I-square index (I^2^) were used to evaluate the statistical heterogeneity and the magnitude of the inconsistency, respectively. The inconsistency was considered high if the I^2^ value was above 50% and low if the value was below 25%. For the meta-analysis, data on MML, Mesial and Distal Papilla Height, and MBL were extracted into Review Manager (Version 5.4.1) [24].

## Results

### Search results

**Fig. 1:**
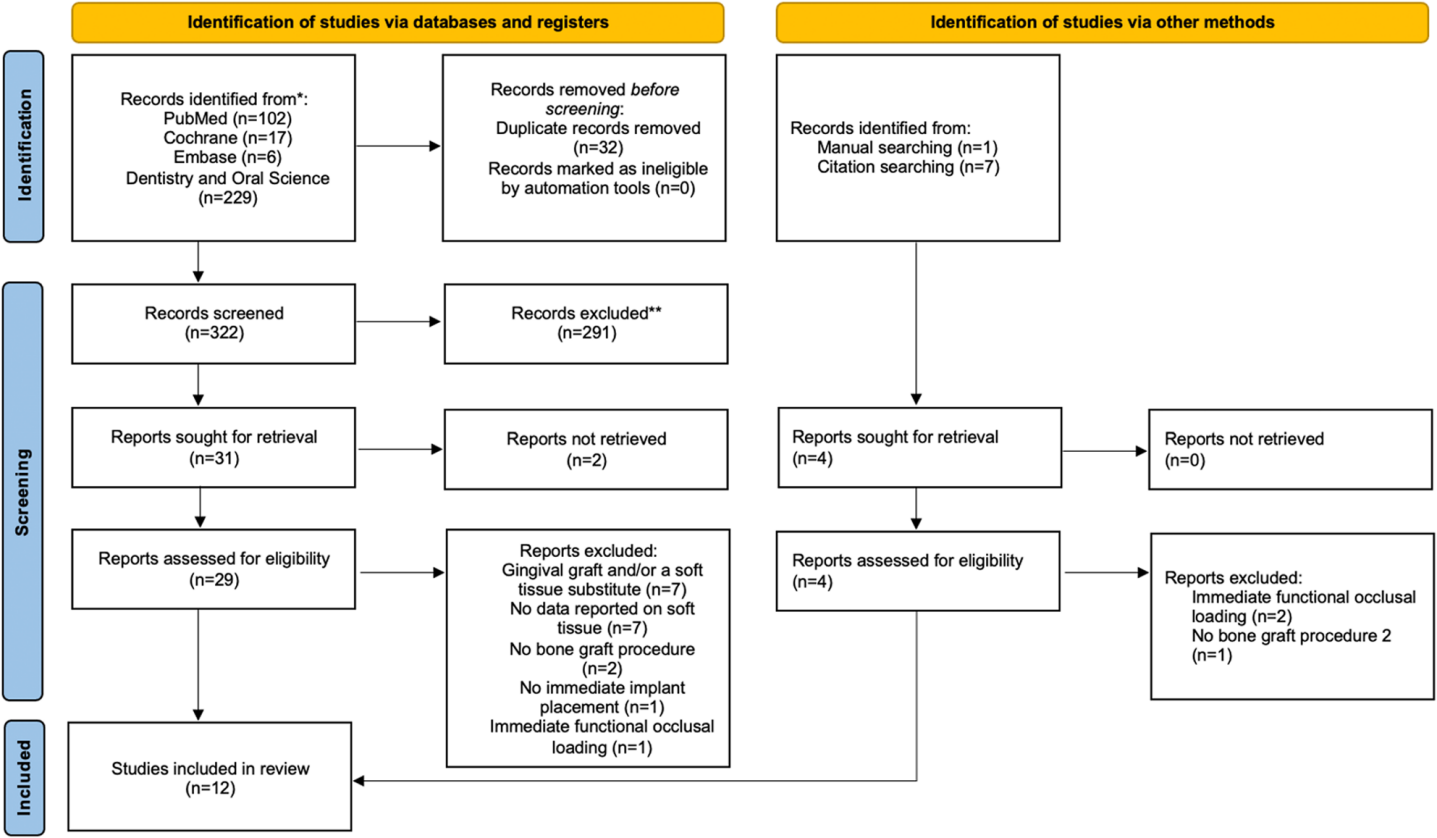
Flow chart for the search and selection process of this systematic review

The electronic and manual search process, depicted in Fig. 1 (flowchart), yielded a total of 362 records, sourced from PubMed (102), Cochrane (17), Embase (6), Dentistry and Oral Science (229), citation searching (7) and manual search (1) (Fig. 1). After excluding 32 duplicates, using Rayyan, records were screened by titles and abstracts, resulting in further exclusions.

Consequently, 35 publications were selected for full-text analysis. Among these, 23 studies were excluded due to various reasons: in seven studies, protocols were combined with a gingival graft and/or a soft tissue substitute; in seven studies, no data were reported on soft tissue; in three studies, there was no bone graft procedure; in one study no immediate implant placement was performed; in three studies, no immediate provisionalization was done; and in two studies there was no access to full text. Consequently, 12 publications remained for analysis (Supplemental Fig. 1). A high level of inter-reviewer agreement (κ = 0.91; 95% CI: [0.78; 1.04]; *p* < 0.001) was observed.

### Risk of bias and quality assessment

The level of evidence of included studies was evaluated using OCEBM Levels of Evidence. This systematic review included 3 studies with a grade of 1B (RCTs), 1 with a grade of 2B (cohort study), 1 with a grade of 3B (case-control) and 6 with a grade of 4 (case-series).

**Fig. 2:**
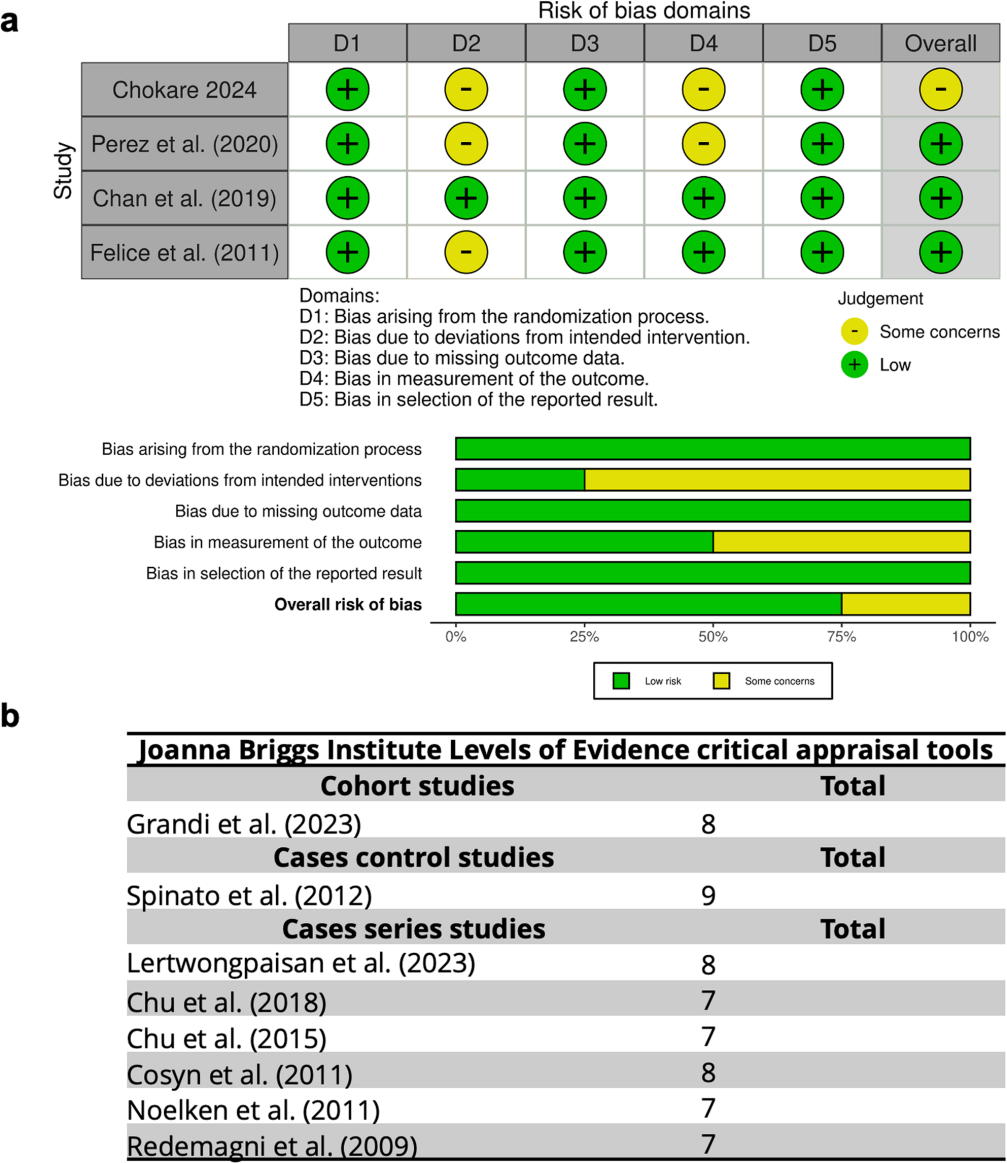
(**a**) Risk of bias for RCTs assessed using the RoB2 tool. (**b**) Risk of bias for cases series, cohort and control cases studies assessed using Joanna Briggs Institute Levels of Evidence critical appraisal tools

RoB for RCTs was assessed using the RoB2 tool (Fig. 2A), which indicated a generally low RoB across the included studies. Some concerns were noted about a possible bias due to deviations from the intended interventions when immediate provisionalization was performed. Indeed, provisional restorations are visible to patients during healing and to examiners when measuring the outcome, it could introduce bias. Moreover, one study highlighted concerns regarding the limited sample size [25]. However, the analysis suggests that these factors did not impact on the overall results.

RoB for cases series, cohort and control cases studies were assessed using Joanna Briggs Institute Levels of Evidence critical appraisal tools (Fig. 2B; Supplemental Fig. 2). Evaluated criteria included inclusion criteria, standardization of measures, follow-up. All studies scored seven or more, indicating a low RoB.

### Characteristics of included studies

Twelve studies were analyzed (Table 1) representing a total of 431 patients and 438 implants. 277 were immediate implants with immediate provisionalization associated with bone grafting. 42% of studies (5 out 12) were conducted in Italy. Supplemental Tables 2–4 provide additional parameters from the included studies not detailed in this review.

**Table 1:** Included articles for the systematic review

Author	Year	Type of study	Protocol description		Mean age (Years)	Number of patients		Number of implants		Follow-up (months)
			Test	Control		Test	Control	Test	Control	
Chokaree	2024	RCT	IIP – BG - customized HA	IIP – BG - standard HA	58,5	6	6	6	6	6
Lertwongpaisan	2023	Case-series	IIP – BG - titanium customized HA	N.A	49,28	30	0	32	0	6
Perez	2020	RCT	IIP – BG - customized HA	IIP – BG - Standard HA	58	18	18	18	18	12
Chan	2019	RCT	IIP – BG - IP	IIP – BG - standard HA	59,08	18	20	18	20	12
Chu	2018	Case series	IIP – BG - IP	IIP – BG - standard HA	48,5	17	6	17	6	5
Chu	2015	Case series	IIP - IP	IIP - standard HA	48,5	17	27	17	28	5
			IIP – BG - standard HA							
			IIP - BG - IP							
Grandi	2013	Cohort study	IIP - BG - IP	Socket preservation - delayed implant - IP	56,88	25	25	25	25	4
Spinato	2012	Case Control	IIP - IP - BG	N.A	42,5	20	21	22	23	12–58
Cosyn	2011	Case series	IIP - IP - BG	N.A	54	30	0	30	0	36
Noelken	2011	Case series	IIP - IP - BG	N.A	43	16	0	16	0	13–36
Felice	2011	RCT	IIP - IP - BG	Socket preservation - delayed implant - IP	49	52	52	54	54	4
Redemagni	2009	Case series	IIP - bone graft - IP	N.A	52,11	28	0	28	0	20,4 (6–50)

*IIP* Immediate Implant Placement,* BG *Bone Graft*, HA *Healing Abutment,* IP *Immediate Provisional* NA *Not Applicable,* RCT *Randomized Clinical Trial

**Fig. 3:**
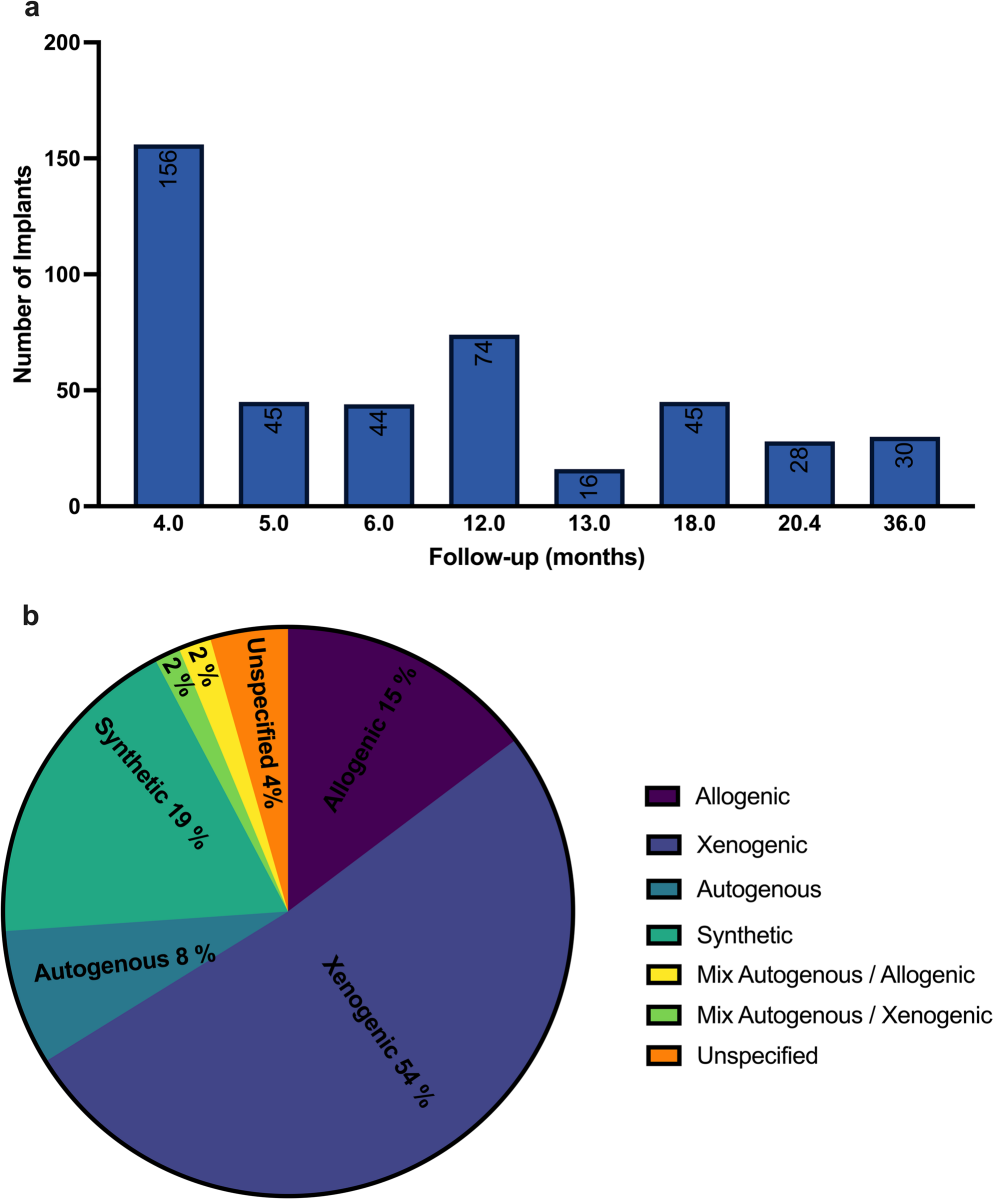
(**a**) Implants follow-up time and (**b**) Type of bone grafting materials

- *Follow-up*: The mean follow-up per implant was 10.6 months; 95% CI: [9.84,11.49; SD: [1.87,19.46] (Fig. 3A). More than 50% of implants were followed only for the duration of osseointegration, which was 6 months or less. Only one study [26] provided a mid-term follow-up at 3 years.
- *Type of bone grafting materials*: The distribution of bone substitute types utilized is as follows (Fig. 3B) : 18% of implants were associated with the use of synthetic bone [27, 28], 15% with allografts [17, 29–31]. 52% with xenogenic bovine bone [26, 30, 32–34], and 8% with autogenous bone [30, 35]. A single study [30] evaluated various bone graft materials and concluded that, in the presence of a thick gingival phenotype, the placement of different graft biomaterials in horizontal gaps does not influence clinical outcomes. This study accounts only for 22 out of 277 immediate implants with bone grafts included in our systematic review. However, the authors were unable to conduct a comparative statistical analysis due to the variability of the grafting materials and their combinations, along with the limited sample size in each group.
- *Initial assessment of hard and soft tissues*: Nine studies exclusively enrolled patients with no initial MBL and presence of the buccal wall of bone. Two studies extended inclusion criteria to patients with up to 4 mm of missing facial plate height [29, 32] One study only included patients with complete loss of the facial bony lamella [35].

Concerning soft tissue requirements, nine studies enrolled only patients with an ideal soft tissue level without any gingival recession compared with contralateral tooth. Two studies specified a thick phenotype as an inclusion criteria [26, 30]. Two studies did not provide specific soft tissue requirements prior to surgery for inclusion. Those two studies did also not require an optimal four walls socket [32, 35].

- *Flap management*: All included studies followed a protocol that did not involve flap elevation.
- *Tooth position*: Most studies (92% of included studies) did an immediate implant placement combined with immediate provisionalization to replace a tooth on anterior region. One study (8%) only performed surgery on maxillary or mandibular molar [28].

### Primary outcome:

**Fig. 4:**
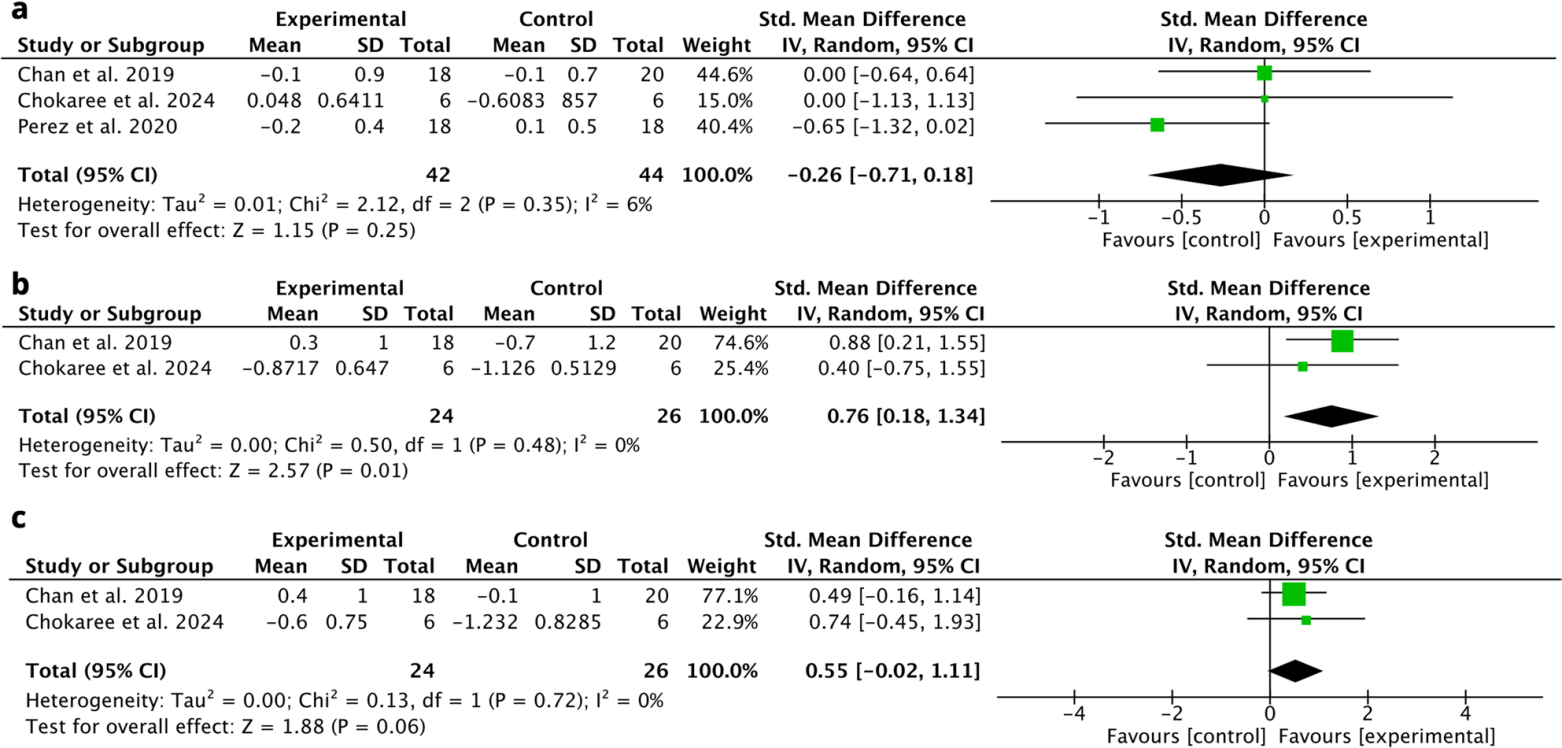
Meta-analysis for (**a**) Midfacial Mucosal Level; (**b**) Mesial Papilla la Height and (**c**) Distal Papilla Height

- *Midfacial Mucosal Level*: The standard mean difference (SMD) for the MML between control and test group was − 0.26; 95%CI: [− 0.71; 0.18; *p* = 0.25] (3 RCTs, 6 to 12 months follow-up), with no statistical difference. The heterogeneity across the studies was low (I^2^ = 6.00%, *p* = 0.35) (Fig. 4A).

MML was also evaluated on a total of six studies: 3 RCTs and 3 non-RCTs [25–30] (Supplemental Table 5). Chan et al. documented limited changes with no significant differences between immediately placed implants with and without immediate provisionalization at different time points. Chokaree et al. observed no significant difference between control group (standard healing abutment) and the test group (customized healing abutment). In contrast, Perez et al. observed significantly less midfacial recession in the control group (standard healing abutment) (0.1 ± 0.5 mm) compared to the test group (customized healing abutment), which exhibited a mean recession of -0.2 ± 0.4 mm. Lertwongpaisan in 2023 reported limited changes of -0,85 mm (± 0,23) but significant alterations between baseline and the 6-month follow-up, with most of the reduction occurring immediately post-extraction and during the first month (-0,79 mm ± 0,23). Spinato et al. (2012) showed no differences between groups (with or without graft). Cosyn et al. (2011) reported stable levels over 3 years.

### Secondary outcome

#### Peri-implant soft tissue parameters

- Papilla Evaluation:

##### Papilla height

The SMD for mesial papilla between control and test group was 0.76; 95%CI: [0.18; 1.34; *p* = 0.01] in favor of the test group. (2 RCTs, 6 to 12 months follow-up). The heterogeneity across the studies was low (I^2^ = 0.00%, *p* = 0.48) (Fig. 4B). Concerning distal papilla, the SMD between control and test group was 0.40; 95%CI: [-0.16; 0.96; *p* = 0.16], with no statistical difference (2 RCTs, 6 to 12 months follow-up). Homogeneity across the studies were observed (I^2^ = 0.00%, *p* = 0.48) [25, 29] (Fig. 4C).

Papilla-related parameters were assessed either through linear measurements of papilla height [26, 29] or using a scoring system [26, 27, 32, 35] such as the Pink Esthetic Score (PES) or the Papilla Index [36] (Supplemental Table 6). Perez et al. reported no statistically significant differences in papilla index between the test and control groups. When evaluated using the PES, papilla scores ranged from 1.44 to 1.85 across studies (with a score of 1 indicating an incomplete papilla and 2 a complete papilla). Overall, the scores appeared generally stable over time and between groups.

- *Peri-implant mucosa thickness*: Facial soft tissue thickness at the gingival level was assessed only by Chu et al. (2015), who compared four different immediate implant placement procedures: with or without bone grafting and with or without immediate provisionalization. Sites that received both a bone graft and a provisional restoration demonstrated greater soft tissue thickness compared to other sites after healing [17].
- *Buccal horizontal alteration*: Chokaree et al. observed minimal linear soft and hard tissue alterations in both groups (standard vs. customized healing abutment) without significant differences. Lertwongpaisan reported also gingival contour shrinkage with a total reduction of 0.70 ± 0.16 mm buccally 6 months after placing a customized titanium healing abutment (Supplemental Table 7).
- *PES*: PES was assessed on 5 studies and achieved a high PES score [25–27, 32, 35]. Studies conducted by Chokaree, Perez, Chan and Felice, reported no difference between the test and control groups. Only Perez and Noelken compared PES scores between baseline and post-operative measurements, and both studies reported no statistically significant differences. (Supplemental Table 8).
- *KMW*: KMW refers to the apical-coronal distance from the gingival crest to the mucogingival junction. Only one study [27] assessed KMW and reported limited changes after healing with no significant differences between the abutment group (utilizing a customized healing abutment) and the control group (standard abutment) (Supplemental Table 9).

#### Peri-implant bone evaluation:

**Fig. 5:**
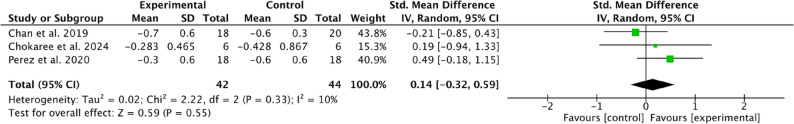
Meta-analysis for Marginal Bone Loss

- *MBL*: The SMD for MBL between control and test group was 0.14; 95%CI: [-0.32; 0.59; *p* = 0.55] with no statistical difference. (3 RCTs, 6 to 12 months follow-up). The heterogeneity across the studies was low (I2 = 10.00%, *p* = 0.55) (Fig. 5).

Seven studies assessed MBL variations (Supplemental Table 10). Chokaree et al. reported no statistical difference in either group at the 6-month at mesial and distal aspects. Perez et al. (2020) observed limited changes in the test group at the 12-month follow-up (0.3 mm ± 0.6 mm) and also found no statistically significant difference between the customized healing abutment group and the control group (standard abutment). Noelken et al. (2011) reported stable marginal bone level around implants. Chan (2019) noted no significant interaction over time or within time points between groups test (single immediately placed implants with immediate provisionalization) and group control (without provisionalization). Spinato (2012) also reported no statistically significant difference between grafted and non-grafted sites, with values of 0.94 mm ± 0.51 mm and 0.90 ± 0.49 mm, respectively.

- *Buccal Bone Thickness*: Only one study assessed peri-implant bone thickness or reduction and found no significant difference between immediately placed implants with and without immediate provisionalization [29].

#### Implant survival rate

Implant survival rates for immediate implants with immediate provisionalization combined with bone grafting were reported in all included studies, ranging from 92% to 100% over follow-up periods spanning from 4 to 58 months. When available, none of the studies reported statistically significant differences between the control and experimental groups (Supplemental Table 11).

## Discussion

This systematic review was designed to evaluate the effect of an open healing approach during immediate implant placement with provisionalization associated with bone grafting on peri-implant soft tissues. A total of twelve studies were analyzed, including four randomized controlled trials (RCTs).

To better understand the outcomes of this approach, the characteristics of the included studies were carefully examined. The varied use of allografts, xenografts, and synthetic bone grafts reflects diverse approaches to achieve optimal osseointegration and tissue response. This diversity makes it challenging to draw definitive conclusions about the superiority of one material over another. The results of the review indicate a trend toward the use of xenografts. Compared with the gold standard autograft, these materials are slowly resorbable and may help to better preserve socket contours and volumes, albeit with less bone content [37].

Most of studies included patients with no alteration of the buccal bone and an ideal soft tissue level. Initial parameters analysis is critical and could potentially influence outcomes related to stability and aesthetic results, which could mean that a careful selection of patients is needed prior to performing immediate placement and more studies are required to validate this protocol when initial situation is degraded. Indeed, this observation aligns with a prior meta-analysis [38] showing that immediate implants in thick phenotype exhibit improved aesthetic outcomes compared to those placed in thin phenotype, specifically regarding mid-facial recession and papilla height maintenance. This highlights the critical role of the initial soft tissue conditions.

All studies included performed flapless surgeries which is in adequation with the bone and soft tissue inclusion criteria that did not require raising a flap for bone augmentation. A single study [35] included only patients with a complete loss of the buccal bone, performed flapless surgery and did not report any major complication. Flapless surgeries offer advantages, which significantly enhance patient outcomes. It minimizes surgical trauma and offers the best possible wound stability, maintains blood supply, which leads to optimize soft tissue healing and reduced scarring [39]. Additionally, these procedures significantly increase patient comfort, allowing individuals to quickly resume their oral hygiene habits soon after the procedure [40].

When assessing midfacial mucosa levels, our meta-analysis showed minimal and acceptable recession between the baseline and healing at 6 to 12 months follow-up. While this could suggest that immediate provisionalization helps to stabilize soft tissue, Chan et al. reported no significant difference when comparing implants placed immediately with and without immediate provisionalization. In contrast, Perez et al. observed less recession in the control group (standard abutments) compared to the test group (customized abutments). Indeed, sealing the socket around a smaller abutment may arithmetically result in a higher gingival level by coronally advancing the flap. In contrast, customized abutments help maintaining the ridge contour, but mucosa level may be more apical. Our findings align with a previous meta-analysis [8] suggesting that immediate provisionalization of immediate implants may support midfacial soft tissue stability. At the end of the treatment, the optimal compromise between mucosal level and ridge contour preservation must be achieved. The maintenance of ridge contour by avoiding buccal concavity and an ideal mucosa level is crucial for both aesthetics and hygiene.

Papilla preservation remains a major challenge, particularly in the esthetic zone. This protocol appears to maintain or even improve papillary outcomes compared to approaches involving primary flap closure. Additionally, the use of provisional restorations designed to replicate the original contour of the natural tooth seems to support papilla height, thereby avoiding its collapse and the need for subsequent reconstruction.

Similarly, the open healing approach did not prevent horizontal buccal alterations. However, this technique was associated with minimal dimensional changes and did not result in any clinically significant drawbacks compared to the control group.

Adequate KMW at the peri-implant site is generally required for long-term stability. A higher prevalence of plaque accumulation, apical migration of the mucosal margin, MBL and peri-implantitis are associated with the absence of keratinized mucosa [41]. Flapless surgery and avoiding a coronally advanced flap to achieve primary closure could be one way to help maintain the position of muco-gingival junction. Here only by one study [27] assessed KMW and confirmed limited changes after healing. Nevertheless, they did not significantly differ between the immediate placement group (utilizing a customized healing abutment) and the control group (standard abutment). This could be partially explained by the small diameter variation between standard and customized abutments given that the surgeries were performed on the anterior maxilla. Therefore, the interaction between restoration design and mucosal response should be considered when interpreting the present findings. Indeed, the design of the prosthetic restoration, particularly the emergence profile, plays a critical role in shaping and maintaining peri-implant soft tissue stability [42, 43]. A convex or over-contoured restoration may exert pressure on the marginal mucosa, potentially leading to recession or mucosal thinning. Conversely, a well-designed, concave profile can promote soft tissue thickening and improved vascularization. Recent studies have highlighted the importance of prosthetic contour in guiding the architecture of the peri-implant mucosa and achieving optimal aesthetic outcomes [44, 45].

Marginal bone loss could not be prevented with this “open healing” approach; however, this approach did not result in significant disadvantages compared with controls. However, given that bone resorption profiles vary depending on jaws region, care must be taken when interpreting these results as most studies were conducted in the anterior area. Buccal bone thickness, which plays a critical role in supporting alveolar width, was assessed in one study [29], which demonstrated minimal peri-implant bone reduction post-healing. We could not conclude whether this open healing approach allows for greater buccal bone thickness, as the study revealed no significant difference between immediately placed implants with and without immediate provisionalization.

Immediate functional occlusal loading was not included in this review and constituted an exclusion criterion. Therefore, the present findings cannot be attributed to differences in mechanical loading conditions but strictly to variations in peri-implant soft tissue management strategies.

Survival rate reported in this systematic review are consistent with current data on both immediate and delayed implants [46, 47], reinforcing the reliability of immediate placement protocols under specific conditions and patient selection criteria. When a control was present, studies reported no significant differences, particularly between immediate and delayed implant placement protocols.

It has long been considered that complete flap closure and primary wound healing is necessary for successful bone graft integration. Currently, open flap procedure with collagen membrane coverage is increasingly used during immediate implants protocol or socket preservation and is able to also achieve favorable bone healing outcomes [48–50]. However, here an open healing of the bone graft protected only by the immediate provisional prosthesis, without a barrier membrane, does not seem to affect the outcome of the peri-implant mucosa. Although no soft tissue complications were reported, more biological and histological analyses are necessary to understand the suitability of bone grafts as scaffolds for soft tissue closure and the impact of encapsulating bone particles on gingival phenotypes. Enhancing our comprehensive understanding of peri-implant mucosa closure is essential, particularly considering the implant site location where the surface area of a molar is significantly larger than that of an anterior tooth, necessitating a more extensive healing process.

To date, only a few authors have highlighted graft particles encapsulated into soft tissue and performed histological analysis [16, 51]. Biomaterial particles were found in the connective tissue in direct contact with collagen fibers, occasionally surrounded by multinucleated cells, whereas the biomaterial particles were occasionally surrounded by newly formed, mineralized bone. Given that a thicker gingival phenotype and more keratinized tissues may contribute to the long-term stability of crestal bone stability around bone-level implants [14, 52, 53], deepening our understanding of peri-implant mucosa phenotype modification and its implications when bone particles are incorporated into connective tissue is crucial.

These findings revealed limitations requiring cautious interpretation. Several articles were excluded because they reported outcomes solely on hard tissues and did not focus on soft tissues. Moreover, we chose to exclude protocols that included gingival grafts to isolate the impact of bone grafts on soft tissues. Given that connective tissue grafting is often recommended in the aesthetic zone, several studies had to be also excluded. Ultimately, only twelve studies were included, with just four being RCTs, the gold standard for establishing causality. The predominance of cohort and case series studies limits the capacity to draw definitive causal inferences regarding the impact of the open healing approach on clinical outcomes, such as soft tissue integration and implant stability. A meta-analysis could not be performed for every outcome because of a lack of data or variability in follow-up times, or heterogeneity in parameter assessment methods. However, the included studies demonstrated a low RoB. Another limitation is the combined analysis of anterior and posterior implant sites, despite potential location-specific differences, due to the limited number of studies available.

This study requires further reinforcement, as high-quality RCTs are still necessary. Subsequently, identifying the biological mechanisms underlying the peri-implant mucosal response to bone particle integration, through the development of relevant in vitro and in vivo models, would enable the optimization of surgical protocols and biomaterial development.

##  Conclusion

The open healing approach, combined with bone grafting during immediate implant placement with provisionalization, represents a viable strategy for maintaining midfacial mucosal architecture and demonstrates favorable stability of both soft and hard tissues. In these selected cases, this minimally invasive technique offers satisfactory clinical outcomes by preserving peri-implant soft and bone tissue volume and minimizing the need for additional surgical procedures.

## Supplementary Information

Supplementary Material 1.

Supplementary Material 2.

Supplementary Material 3.

Supplementary Material 4.

Supplementary Material 5.

Supplementary Material 6.

Supplementary Material 7.

Supplementary Material 8.

Supplementary Material 9.

Supplementary Material 10.

Supplementary Material 11.

Supplementary Material 12.

Supplementary Material 13.

## Data Availability

All data supporting the findings of this study are available within the paper and its Supplementary Information.

## Abbreviations

MML: Midfacial Mucosal Level
MBL: Marginal Bone Loss
PES: Pink Esthetic Score
KMW: Keratinized Mucosa Width
BBT: Buccal Bone Thickness
RCT: Randomized Clinical Trial
RoB: Risk of Bias
OCEBM: Oxford Centre for Evidence-Based Medicine
SMD: Standard mean difference
IIP: Immediate Implant Placement
BG: Bone Graft
HA: Healing Abutment
IP: Immediate Provisional
NA: Not Applicable
