# Supplementary material for: Impact of an open healing approach on peri-implant mucosa following immediate implant placement with transmucosal provisionalization: a systematic review and meta-analysis
Source: BMC Oral Health. 2026 Mar 20;26:759. doi: 10.1186/s12903-026-08105-z (PMC13126965; doi:10.1186/s12903-026-08105-z)
Supplement: Supplementary file 3 — Supplementary Material 3. [file 12903_2026_8105_MOESM3_ESM.docx]

Supplemental Table 1: Research Equation

Research equation PubMed (Medline)

| PubMed/Medline research |
| --- |
| ((immediate dental implant loading [MeSH Terms]) OR (immediate implant placement) OR (immediate loading)) AND ((bone regeneration[MeSH Terms]) OR (bone substitute[MeSH Terms]) OR (bone substitute[MeSH Terms]) OR (guided bone regeneration)) AND ((oral mucosa[MeSH Terms]) OR (gingiva)) |

Research equation Cochrane

| Cochrane Research | |  |  |  |
| --- | --- | --- | --- | --- |
| #1 | MeSH descriptor: [Immediate Dental Implant Loading] explode all trees | | | |
| #2 | MeSH descriptor: [Bone Regeneration] explode all trees | | | |
| #3 | MeSH descriptor: [Bone Substitutes] explode all trees | | | |
| #4 | MeSH descriptor: [Gingiva] explode all trees | | | |
| #5 | MeSH descriptor: [Bone Transplantation] explode all trees | | | |
| #6 | #2 OR #3 OR #5 |  |  |  |
| #7 | #1 AND #4 AND #6 | |  |  |
|  |  |  |  |  |

Research equation Embase

| Embase Research |  |  |  |  |  |  |  |  |  |  |  |  |
| --- | --- | --- | --- | --- | --- | --- | --- | --- | --- | --- | --- | --- |
| ('immediate implant placement'/exp OR 'immediate implant placement') AND ('guided bone regeneration'/exp OR 'guided bone regeneration') AND ('mouth mucosa'/exp OR 'mouth mucosa') | | | | | | | | | | | | |

Research equation Dentistry and Oral science Source

| Dentistry and Oral Science Source |  |  |  |  |  |
| --- | --- | --- | --- | --- | --- |
| (immediate implant placement) AND (bone regeneration) AND (oral mucosa) | | | | | |
