# Supplementary material for: Impact of an open healing approach on peri-implant mucosa following immediate implant placement with transmucosal provisionalization: a systematic review and meta-analysis
Source: BMC Oral Health. 2026 Mar 20;26:759. doi: 10.1186/s12903-026-08105-z (PMC13126965; doi:10.1186/s12903-026-08105-z)
Supplement: Supplementary file 4 — Supplementary Material 4. [file 12903_2026_8105_MOESM4_ESM.docx]

| **Author** | **Year** | **Probing Pocket Depth** | | | | | | | | | | | | | | | | | | | | | | | | | | |
| --- | --- | --- | --- | --- | --- | --- | --- | --- | --- | --- | --- | --- | --- | --- | --- | --- | --- | --- | --- | --- | --- | --- | --- | --- | --- | --- | --- | --- |
|  |  | **Test** | | | | | | | | | | | | | | | **Control** | | | | | | | | | | | |
|  |  | **1 year** | | | | | | | | **3 years** | | | | | | | **1 year** | | | | | | **3 years** | | | | | |
|  |  | **Mean** | **SD** | **Mesial** | **SD** | **Mid** | **SD** | **Distal** | **SD** | **Mean** | **SD** | **Mesial** | **SD** | **Mid** | **SD** | **Distal** | **SD** | **Mesial** | **SD** | **Mid** | **SD** | **Distal** | **SD** | **Mesial** | **SD** | **Mid** | **SD** | **Distal** |
| Spinato et al. | 2012 | NA | NA | 3.5 | 0.8 | 2.8 | 1.1 | 3.6 | 0.7 | NA | NA | NA | NA | NA | NA | NA | NA | 3.2 | 0.6 | 2.3 | 0.6 | 3.5 | 0.8 | NA | NA | NA | NA | NA |
| Cosyn et al. | 2011 | 3.46 | 0.69 | NA | NA | NA | NA | NA | NA | 3.17 | 0.63 | NA | NA | NA | NA | NA | NA | NA | NA | NA | NA | NA | NA | NA | NA | NA | NA | NA |

Supplemental Table 3 : Probing Pocket Depth
