# Supplementary material for: Impact of an open healing approach on peri-implant mucosa following immediate implant placement with transmucosal provisionalization: a systematic review and meta-analysis
Source: BMC Oral Health. 2026 Mar 20;26:759. doi: 10.1186/s12903-026-08105-z (PMC13126965; doi:10.1186/s12903-026-08105-z)
Supplement: Supplementary file 5 — Supplementary Material 5. [file 12903_2026_8105_MOESM5_ESM.docx]

Supplemental Table 2 : Origin of studies and description of implants.

| **Author** | **Year** | **Type of study** | **Country** | **University / Private** | **Implant** | |  |
| --- | --- | --- | --- | --- | --- | --- | --- |
|  |  |  |  |  | **Type of Implant** | **Brand** |  |
| Chokaree et al. | 2024 | RCT | Thailand | University | BL / IC | Neobiotech |  |
| Lertwongpaisan et al. | 2023 | Case-series | Thailand | University | BL / IC | Dentium |  |
| Perez et al. | 2020 | RCT | Switzerland/ Italy | University | BL / IC | Strauman |  |
| Chan et al. | 2019 | RCT | USA | University | BL / IC | Neobiotech |  |
| Chu et al. | 2018 | Case series | USA | University | BL | Unspecified |  |
| Chu et al. | 2015 | Case series | USA | University | BL / IC | Unknow |  |
|  |  |  |  |  |  |  |  |
|  |  |  |  |  |  |  |  |
| Grandi et al. | 2013 | Cohort study | Italy | Private | BL / IC | JDentalCare |  |
| Spinato et al. | 2012 | Case Control | Italy | Private | BL / IC | Zimmer Dental |  |
| Cosyn et al. | 2011 | Case series | Belgium | University | BL / IC | Nobel Biocare |  |
| Noelken et al. | 2011 | Case series | Germany | Private | BL / EC | Nobel Biocare |  |
| Felice et al. | 2011 | RCT | Italy | Private | BL / IC | MegaGen |  |
| Redemagni et al. | 2009 | Case series | Italy | Private | BL / IC | Denstpy |  |
