# Supplementary material for: Impact of an open healing approach on peri-implant mucosa following immediate implant placement with transmucosal provisionalization: a systematic review and meta-analysis
Source: BMC Oral Health. 2026 Mar 20;26:759. doi: 10.1186/s12903-026-08105-z (PMC13126965; doi:10.1186/s12903-026-08105-z)
Supplement: Supplementary file 6 — Supplementary Material 6. [file 12903_2026_8105_MOESM6_ESM.docx]

| **Author** | **Year** | **Type of study** | **Protocol description** | | **Number of implants** | | **Midfacial Mucosal Level** | | | | | | | | | | | | | | | |
| --- | --- | --- | --- | --- | --- | --- | --- | --- | --- | --- | --- | --- | --- | --- | --- | --- | --- | --- | --- | --- | --- | --- |
|  |  |  | **Test** | **Control** | **Test** | **Control** | **Test** | | | | | | | | **Contrôle** | | | | | | | |
|  |  |  |  |  |  |  | **0-3 months** | | **0-6 months** | | **0-12 months** | | **0-36 months** | | **0-1 month** | | **0-3 months** | | **0-6 months** | | **0-12 months** | |
|  |  |  |  |  |  |  | **Mean** | **SD** | **Mean** | **SD** | **Mean** | **SD** | **Mean** | **SD** | **Mean** | **SD** | **Mean** | **SD** | **Mean** | **SD** | **Mean** | **SD** |
| Chokaree et al. | 2024 | RCT | IIP – BG - customized HA - | IIP – BG - Standard HA | 6 | 6 | NA | NA | 0.048 | 0.6411 | NA | NA | NA | NA | NA | NA | NA | NA | -0.6083 | 0.857 | NA | NA |
| Lertwongpaisan et al. | 2023 | Case-series | IIP – BG - titanium customized HA - | N.A | 32 | 0 | 0.04 | 0.13 | -0.22 | 0.16 | NA | NA | NA | NA | NA | NA | NA | NA | NA | NA | NA | NA |
| Perez et al. | 2020 | RCT | IIP – BG - customized HA | IIP – BG - Standard HA | 18 | 18 | NA | NA | NA | NA | -0.2 | 0.4 | NA | NA | NA | NA | NA | NA | NA | NA | +0.1 | 0.5 |
| Chan et al. | 2019 | RCT | IIP – BG - IP | IIP – BG - standard HA | 18 | 20 | NA | NA | NA | NA | -0.1 | 0.9 | NA | NA | NA | NA | NA | NA | NA | NA | -0.1 | 0.7 |
| Spinato et al. | 2012 | Case Control | IIP - IP - BG | IIP - IP | 22 | 23 | NA | NA | NA | NA | -0.23 | 0.52 | NA | NA | NA | NA | NA | NA | NA | NA | -0.15 | 0.32 |
| Cosyn et al. | 2011 | Case series | IIP - IP - BG | N.A | 30 | 0 | NA | NA | NA | NA | -0.53 | 0.76 | -0.34 | 0.8 | NA | NA | NA | NA | NA | NA | NA | NA |
| *Negative values indicate recession or dimensional reduction.*  *IIP: Immediate Implant Placement; BG: Bone Graft; HA: Healing Abutment; IP: Immediate Provisional; NA: Not Applicable; RCT : Randomized Clinical Trial; BL : Bone Level ; IC : Internal Connection ; EC : External Connection* | | | | | | | | | | | | | | | | | | | | | | |

Supplemental Table 5: Midfacial Mucosal Level
