# Supplementary material for: Impact of an open healing approach on peri-implant mucosa following immediate implant placement with transmucosal provisionalization: a systematic review and meta-analysis
Source: BMC Oral Health. 2026 Mar 20;26:759. doi: 10.1186/s12903-026-08105-z (PMC13126965; doi:10.1186/s12903-026-08105-z)
Supplement: Supplementary file 7 — Supplementary Material 7. [file 12903_2026_8105_MOESM7_ESM.docx]

| **Author** | **Year** | **Plaque Score (%)** | | | | **Bleeding On Probing (%)** | | | |
| --- | --- | --- | --- | --- | --- | --- | --- | --- | --- |
|  |  | **Test** | | | | **Test** | | | |
|  |  | **1 year** | | **3 Years** | | **1 year** | | **3 Years** | |
|  |  | **Mean** | **SD** | **Mean** | **SD** | **Mean** | **SD** | **Mean** | **SD** |
| Spinato et al. | 2012 | NA | NA | NA | NA | NA | NA | NA | NA |
| Cosyn et al. | 2011 | 17 | 18 | 18 | 17 | 41 | 16 | 24 | 19 |

Supplemental Table 4 : Plaque Score and Bleeding on Probing
