# Supplementary material for: Impact of an open healing approach on peri-implant mucosa following immediate implant placement with transmucosal provisionalization: a systematic review and meta-analysis
Source: BMC Oral Health. 2026 Mar 20;26:759. doi: 10.1186/s12903-026-08105-z (PMC13126965; doi:10.1186/s12903-026-08105-z)
Supplement: Supplementary file 8 — Supplementary Material 8. [file 12903_2026_8105_MOESM8_ESM.docx]

| **Author** | **Year** | **Type of study** | **Protocol description** | | **Number of implants** | | **Width of keratinized tissue** | | | | | | | | | | | |
| --- | --- | --- | --- | --- | --- | --- | --- | --- | --- | --- | --- | --- | --- | --- | --- | --- | --- | --- |
|  |  |  | **Test** | **Control** | **Test** | **Control** | **Test** | | | | | | **Contrôle** | | | | | |
|  |  |  |  |  |  |  | **0** | | **4 months** | | **12 months** | | **0** | | **4 months** | | **12 months** | |
|  |  |  |  |  |  |  | **Mean** | **SD** | **Mean** | **SD** | **Mean** | **SD** | **Mean** | **SD** | **Mean** | **SD** | **Mean** | **SD** |
| Perez et al. | 2020 | RCT | IIP – BG - customized HA | IIP – BG - Standard HA | 18 | 18 | 4.2 | 1.3 | 4 | 1 | 4 | 1.1 | 3.5 | 1.2 | 3.3 | 1 | 3.3 | 0.8 |

Supplemental Table 9 : Width of keratinized tissue
