# Supplementary material for: Impact of an open healing approach on peri-implant mucosa following immediate implant placement with transmucosal provisionalization: a systematic review and meta-analysis
Source: BMC Oral Health. 2026 Mar 20;26:759. doi: 10.1186/s12903-026-08105-z (PMC13126965; doi:10.1186/s12903-026-08105-z)
Supplement: Supplementary file 9 — Supplementary Material 9. [file 12903_2026_8105_MOESM9_ESM.docx]

| **Author** | **Year** | **Type of study** | **Protocol description** | | **Number of implants** | | **PES** | | | | | | | | | | | | | | | |
| --- | --- | --- | --- | --- | --- | --- | --- | --- | --- | --- | --- | --- | --- | --- | --- | --- | --- | --- | --- | --- | --- | --- |
|  |  |  | **Test** | **Control** | **Test** | **Control** | **Test** | | | | | | | | **Control** | | | | | | | |
|  |  |  |  |  |  |  | **4 months** | | **0-6 months (changes)** | | **1 year** | | **3 year** | | **4 months** | | **0-6 months (changes)** | | **1 year** | | **3 year** | |
|  |  |  |  |  |  |  | **Mean** | **SD** | **Mean** | **SD** | **Mean** | **SD** | **Mean** | **SD** | **Mean** | **SD** | **Mean** | **SD** | **Mean** | **SD** | **Mean** | **SD** |
| Chokaree et al. | 2024 | RCT | IIP – BG - customized HA - | IIP – BG - Standard HA | 6 | 6 | NA | NA | −0.333 | 1.51 | NA | NA | NA | NA | NA | NA | -2.75 | 0.96 | NA | NA | NA | NA |
| Perez et al. | 2020 | RCT | IIP – BG - customized HA | IIP – BG - Standard HA | 18 | 18 | NA | NA | NA | NA | 8,7 | 1 | NA | NA | NA | NA | NA | NA | 8.2 | 0.7 | NA | NA |
| Cosyn et al. | 2011 | Case series | IIP - IP - BG | N.A | 30 | 0 | NA | NA | NA | NA | NA | NA | 10,48 | 2,47 | NA | NA | NA | NA | NA | NA | NA | NA |
| Noelken et al. | 2011 | Case series | IIP - IP - BG | N.A | 16 | 0 | NA | NA | NA | NA | 12,5 | 1,25 | NA | NA | NA | NA | NA | NA | NA | NA | NA | NA |
| Felice et al. | 2011 | RCT | IIP - IP - BG | Socket preservation - delayed implant - IP | 54 | 54 | 12,75 | 1,25 | NA | NA | NA | NA | NA | NA | 12.62 | 1.05 | NA | NA | NA | NA | NA | NA |
| *IIP: Immediate Implant Placement; BG: Bone Graft; HA: Healing Abutment; IP: Immediate Provisional; NA: Not Applicable; RCT : Randomized Clinical Trial; BL : Bone Level ; IC : Internal Connection ; EC : External Connection* | | | | | | | | | | | | | | | | | | | | | | |

Supplemental Table 8: Pink Esthetic Score
