# Supplementary material for: Impact of an open healing approach on peri-implant mucosa following immediate implant placement with transmucosal provisionalization: a systematic review and meta-analysis
Source: BMC Oral Health. 2026 Mar 20;26:759. doi: 10.1186/s12903-026-08105-z (PMC13126965; doi:10.1186/s12903-026-08105-z)
Supplement: Supplementary file 10 — Supplementary Material 10. [file 12903_2026_8105_MOESM10_ESM.docx]

| **Author** | **Year** | **Papilla Height** | | | | | | | | | | | | **Papilla Index** | | | | | | | | | | | | | | | |
| --- | --- | --- | --- | --- | --- | --- | --- | --- | --- | --- | --- | --- | --- | --- | --- | --- | --- | --- | --- | --- | --- | --- | --- | --- | --- | --- | --- | --- | --- |
|  |  | **Test** | | | | | | | | **Contrôle** | | | | **Test** | | | | | | | | **Contrôle** | | | | | | | |
|  |  | **0-12 months** | | | | **0-36 months** | | | | **0-12 months** | | | | **0-4 months** | | | | **0-12 months** | | | | **0-4 months** | | | | **0-12 months** | | | |
|  |  | **Mesial Mean** | **SD** | **Distal Mean** | **SD** | **Mesial Mean** | **SD** | **Distal Mean** | **SD** | **Mesial Mean** | **SD** | **Distal Mean** | **SD** | **Mesial Mean** | **SD** | **Distal Mean** | **SD** | **Mesial Mean** | **SD** | **Distal Mean** | **SD** | **Mesial Mean** | **SD** | **Distal Mean** | **SD** | **Mesial Mean** | **SD** | **Distal Mean** | **SD** |
| Perez et al. | 2020 | NA | NA | NA | NA | NA | NA | NA | NA | NA | NA | NA | NA | 2.2 | 0.6 | 2 | 0.8 | 2.4 | 0.5 | 2.6 | 0.6 | 1.6 | 0.5 | 1.1 | 0.8 | 2.0 | 0.5 | 1.6 | 0.6 |
| Chan et al. | 2019 | 0.3 | 1 | 0.4 | 1 | NA | NA | NA | NA | -0.7 | 1.2 | 0.1 | 1 | NA | NA | NA | NA | NA | NA | NA | NA | NA | NA | NA | NA | NA | NA | NA | NA |
| Cosyn et al. | 2011 | -0.41 | 0.71 | -0.31 | 0.83 | -0.05 | 0.83 | -0.08 | 1.24 | NA | NA | NA | NA | NA | NA | NA | NA | NA | NA | NA | NA | NA | NA | NA | NA | NA | NA | NA | NA |
| Noelken et al. | 2011 | NA | NA | NA | NA | NA | NA | NA | NA | NA | NA | NA | NA | NA | NA | NA | NA | NA | NA | NA | NA | NA | NA | NA | NA | NA | NA | NA | NA |
| Felice et al. | 2011 | NA | NA | NA | NA | NA | NA | NA | NA | NA | NA | NA | NA | NA | NA | NA | NA | NA | NA | NA | NA | NA | NA | NA | NA | NA | NA | NA | NA |

| **Author** | **Year** | **Papilla (via PES score)** | | | | | | | | | | | | | | | |
| --- | --- | --- | --- | --- | --- | --- | --- | --- | --- | --- | --- | --- | --- | --- | --- | --- | --- |
|  |  | **Test** | | | | | | | | | | | | **Control** | | | |
|  |  | **4 months** | | | | **1 year** | | | | **3 years** | | | | **4 months** | | | |
|  |  | **Mesial Mean** | **SD** | **Distal Mean** | **SD** | **Mesial Mean** | **SD** | **Distal Mean** | **SD** | **Mesial Mean** | **SD** | **Distal Mean** | **SD** | **Mesial Mean** | **SD** | **Distal Mean** | **SD** |
| Perez et al. | 2020 | NA | NA | NA | NA | NA | NA | NA | NA | NA | NA | NA | NA | NA | NA | NA | NA |
| Chan et al. | 2019 | NA | NA | NA | NA | NA | NA | NA | NA | NA | NA | NA | NA | NA | NA | NA | NA |
| Cosyn et al. | 2011 | NA | NA | NA | NA | NA | NA | NA | NA | 1.44 | 0.65 | 1.36 | 0.76 | NA | NA | NA | NA |
| Noelken et al. | 2011 | NA | NA | NA | NA | 1.6 | NA | 1.7 | NA | NA | NA | NA | NA | NA | NA | NA | NA |
| Felice et al. | 2011 | 1.85 | 0.36 | 1.75 | 0.44 | NA | NA | NA | NA | NA | NA | NA | NA | 1.75 | 0.44 | 1.73 | 0.45 |
| *Negative values indicate recession or dimensional reduction.*  *IIP: Immediate Implant Placement; BG: Bone Graft; HA: Healing Abutment; IP: Immediate Provisional; NA: Not Applicable; RCT : Randomized Clinical Trial; BL : Bone Level ; IC : Internal Connection ; EC : External Connection ; PES : Pink Esthetic Score* | | | | | | | | | | | | | | | | | |

Supplemental Table 6 : Papilla evaluation of included studies via Papilla Height, Papilla Index and PES score
