# Supplementary material for: Impact of an open healing approach on peri-implant mucosa following immediate implant placement with transmucosal provisionalization: a systematic review and meta-analysis
Source: BMC Oral Health. 2026 Mar 20;26:759. doi: 10.1186/s12903-026-08105-z (PMC13126965; doi:10.1186/s12903-026-08105-z)
Supplement: Supplementary file 11 — Supplementary Material 11. [file 12903_2026_8105_MOESM11_ESM.docx]

| **Author** | **Year** | **Type of study** | **Implant Survival Rate / Insertion torque** | | | | | | | | | | | | | | | | | | | | |
| --- | --- | --- | --- | --- | --- | --- | --- | --- | --- | --- | --- | --- | --- | --- | --- | --- | --- | --- | --- | --- | --- | --- | --- |
|  |  |  | **Test** | | | | | | | | | | **Contrôle** | | | | | | | | | | |
|  |  |  | **4 months** | | | **6 months** | | | **12 months** | | | | **4 months** | | | | **12 months** | | | | **36 months** | | |
|  |  |  | **Mean** | **Torque** | **Torque (SD)** | **Mean** | **Torque** | **Torque (SD)** | **Mean** | **Torque** | **Torque (SD)** | **Mean** | | **Torque** | **Torque (SD)** | **Mean** | | **Torque** | **Torque (SD)** | **Mean** | | **Torque** | **Torque (SD)** |
| Lertwongpaisan et al. | 2023 | Case-series | NA | NA | NA | 100 | NA | NA | NA | NA | NA | NA | | NA | NA | NA | | NA | NA | NA | | NA | NA |
| Perez et al. | 2020 | RCT | NA | NA | NA | NA | NA | NA | 100 | 37.9 | 12.1 |  | | NA | NA | -0.2 | | 39.9 | 10.8 |  | | NA | NA |
| Chan et al. | 2019 | RCT | NA | NA | NA | NA | NA | NA | 90 | NA | NA | NA | | NA | NA | 100 | | NA | NA | NA | | NA | NA |
| Chu et al. | 2018 | Case series | NA | NA | NA | NA | NA | NA | 100 | NA |  |  | | NA | NA | 100 | | NA | NA |  | | NA | NA |
| Chu et al. | 2015 | Case series | NA | NA | NA | NA | NA | NA | 100 | NA | NA | NA | | NA | NA | 100 | | NA | NA | NA | | NA | NA |
|  |  |  |  |  |  |  |  |  |  |  |  |  |  |  |  |  |  |  |  |  |  |  |  |
|  |  |  |  |  |  |  |  |  |  |  |  |  |  |  |  |  |  |  |  |  |  |  |  |
| Grandi et al. | 2013 | Cohort study | NA | NA | NA | NA | NA | NA | 92 | 72.2 | 11.73 | NA | | NA | NA | 96 | | 62.4 | 11.65 | NA | | NA | NA |
| Spinato et al. | 2012 | Case Control | NA | NA | NA | NA | NA | NA | 100 | NA | NA | NA | | NA | NA | 100 | | NA | NA | NA | | NA | NA |
| Cosyn et al. | 2011 | Case series | NA | NA | NA | NA | NA | NA | NA | NA | NA | NA | | NA | NA | NA | | NA | NA | 96 | | NA | NA |
| Noelken et al. | 2011 | Case series | NA | NA | NA | NA | NA | NA | 100 | NA | NA | NA | | NA | NA | NA | | NA | NA | NA | | NA | NA |
| Felice et al. | 2011 | RCT | 96 | NA | NA | NA | NA | NA | NA | NA | NA | 100 | | NA | NA | NA | | NA | NA | NA | | NA | NA |
| Redemagni et al. | 2009 | Case series | NA | NA | NA | 100 | NA | NA | NA | NA | NA | NA | | NA | NA | NA | | NA | NA | NA | | NA | NA |
| *IIP: Immediate Implant Placement; BG: Bone Graft; HA: Healing Abutment; IP: Immediate Provisional; NA: Not Applicable; RCT : Randomized Clinical Trial; BL : Bone Level ; IC : Internal Connection ; EC : External Connection* | | | | | | | | | | | | | | | | | | | | | | | |

Supplemental Table 11 : Implant Survival Rate and Insertion Torque.
