# Supplementary material for: Impact of an open healing approach on peri-implant mucosa following immediate implant placement with transmucosal provisionalization: a systematic review and meta-analysis
Source: BMC Oral Health. 2026 Mar 20;26:759. doi: 10.1186/s12903-026-08105-z (PMC13126965; doi:10.1186/s12903-026-08105-z)
Supplement: Supplementary file 12 — Supplementary Material 12. [file 12903_2026_8105_MOESM12_ESM.docx]

| **Author** | **Year** | **Marginal Bone Loss** | | | | | | | | | | | | | | | | | | | | | | | |
| --- | --- | --- | --- | --- | --- | --- | --- | --- | --- | --- | --- | --- | --- | --- | --- | --- | --- | --- | --- | --- | --- | --- | --- | --- | --- |
|  |  | **Test** | | | | | | | | | | | | | | | | | | | | | | | |
|  |  | **0-4 months** | | | | | | **0-6 months** | | | | | | **0-12 months** | | | | | | **0-36 months** | | | | | |
|  |  | **Mean** | **SD** | **Mesial Mean** | **SD** | **Distal Mean** | **SD** | **Mean** | **SD** | **Mesial Mean** | **SD** | **Distal Mean** | **SD** | **Mean** | **SD** | **Mesial Mean** | **SD** | **Distal Mean** | **SD** | **Mean** | **SD** | **Mesial Mean** | **SD** | **Distal Mean** | **SD** |
| Chokaree et al. | 2024 | NA | NA | NA | NA | NA | NA | NA | NA | -0.283 | 0.465 | -0.359 | 1.274 | NA | NA | NA | NA | NA | NA | NA | NA | NA | NA | NA | NA |
| Perez et al. | 2020 | 0 | 0.4 | -0.1 | 0.5 | 0.1 | 0.4 | NA | NA | NA | NA | NA | NA | -0.3 | 0.6 | 0 | 0.6 | -0.6 | 0.7 | NA | NA | NA | NA | NA | NA |
| Chan et al. | 2019 | NA | NA | NA | NA | NA | NA | NA | NA | NA | NA | NA | NA | -0.7 | 0.6 | NA | NA | NA | NA | NA | NA | NA | NA | NA | NA |
| Grandi et al. | 2013 | NA | NA | NA | NA | NA | NA | NA | NA | NA | NA | NA | NA | -0.71 | (95% CI 0.45, 0.97) i | NA | NA | NA | NA | NA | NA | NA | NA | NA | NA |
| Spinato et al. | 2012 | NA | NA | NA | NA | NA | NA | NA | NA | NA | NA | NA | NA | -0.65 | 0.52 | NA | NA | NA | NA | NA | NA | NA | NA | NA | NA |
| Cosyn et al. | 2011 | NA | NA | NA | NA | NA | NA | NA | NA | NA | NA | NA | NA | NA | NA | -0.98 | -0.5 | -0.78 | -0.55 | NA | NA | -1.13 | -0.43 | -0.86 | -0.54 |
| Redemagni et al. | 2009 | NA | NA | NA | NA | NA | NA | NA | NA | -0.28 | (-1; 0.5) | -0.24 | (-1; 0.5) | NA | NA | NA | NA | NA | NA | NA | NA | NA | NA | NA | NA |

| **Author** | **Year** | **Marginal Bone Loss** | | | | | | | | | | | | | | | | | |
| --- | --- | --- | --- | --- | --- | --- | --- | --- | --- | --- | --- | --- | --- | --- | --- | --- | --- | --- | --- |
|  |  | **Contrôle** | | | | | | | | | | | | | | | | | |
|  |  | **0-4 months** |  |  |  |  |  | **0-6 months** |  |  |  |  |  | **0-12 months** |  |  |  |  |  |
|  |  | **Mean** | **SD** | **Mesial Mean** | **SD** | **Distal Mean** | **SD** | **Mean** | **SD** | **Mesial Mean** | **SD** | **Distal Mean** | **SD** | **Mean** | **SD** | **Mesial Mean** | **SD** | **Distal Mean** | **SD** |
| Chokaree et al. | 2024 | NA | NA | NA | NA | NA | NA | NA | NA | -0.428 | 0.867 | -0.158 | 0.353 | NA | NA | NA | NA | NA | NA |
| Perez et al. | 2020 | -0.2 | 0.3 | -0.2 | 0.4 | -0.2 | 0.3 | NA | NA | NA | NA | NA | NA | -0.6 | 0.3 | -0.6 | 0.4 | -0.6 | 0.4 |
| Chan et al. | 2019 | NA | NA | NA | NA | NA | NA | NA | NA | NA | NA | NA | NA | -0.6 | 0.6 | NA | NA | NA | NA |
| Grandi et al. | 2013 | NA | NA | NA | NA | NA | NA | NA | NA | NA | NA | NA | NA | -0.60 | 95% CI 0.38, 0.82 | NA | NA | NA | NA |
| Spinato et al. | 2012 | NA | NA | NA | NA | NA | NA | NA | NA | NA | NA | NA | NA | -0.55 | 0.38 | NA | NA | NA | NA |
| Cosyn et al. | 2011 | NA | NA | NA | NA | NA | NA | NA | NA | NA | NA | NA | NA | NA | NA | NA | NA | NA | NA |
| Redemagni et al. | 2009 | NA | NA | NA | NA | NA | NA | NA | NA | NA | NA | NA | NA | NA | NA | NA | NA | NA | NA |
| \| *Negative values indicate recession or dimensional reduction.*  *IIP: Immediate Implant Placement; BG: Bone Graft; HA: Healing Abutment; IP: Immediate Provisional; NA: Not Applicable; RCT : Randomized Clinical Trial; BL : Bone Level ; IC : Internal Connection ; EC : External Connection ; PES : Pink Esthetic Score* \| \| --- \| | | | | | | | | | | | | | | | | | | | |

Supplemental Table 10: Marginal Bone Loss
