# Supplementary material for: Impact of an open healing approach on peri-implant mucosa following immediate implant placement with transmucosal provisionalization: a systematic review and meta-analysis
Source: BMC Oral Health. 2026 Mar 20;26:759. doi: 10.1186/s12903-026-08105-z (PMC13126965; doi:10.1186/s12903-026-08105-z)
Supplement: Supplementary file 13 — Supplementary Material 13. [file 12903_2026_8105_MOESM13_ESM.docx]

| **Author** | **Year** | **Type of study** | **Protocol description** | | **Number of implants** | | **Buccal horizontal alteration** | | | | | | | | | | | | | |
| --- | --- | --- | --- | --- | --- | --- | --- | --- | --- | --- | --- | --- | --- | --- | --- | --- | --- | --- | --- | --- |
|  |  |  | **Test** | **Control** | **Test** | **Control** | **Test** | | | | | | | | **Contrôle** | | | | | |
|  |  |  |  |  |  |  | **0-1 month** | | **0-3 months** | | **0-4 months** | | **0-6 months** | | **0-1 month** | | **0-4 months** | | **0-6 months** | |
|  |  |  |  |  |  |  | **Mean** | **SD** | **Mean** | **SD** | **Mean** | **SD** | **Mean** | **SD** | **Mean** | **SD** | **Mean** | **SD** | **Mean** | **SD** |
| Chokaree et al. | 2024 | RCT | IIP – BG - customized HA - | IIP – BG - Standard HA | 6 | 6 | -0.0754 | 0.509 | NA | NA | -0.5388 | 0.340 | -0.3507 | 0.439 | -0.442 | 0.537 | -0.490 | 0.587 | -0.453 | 0.578 |
| Lertwongpaisan et al. | 2023 | Case-series | IIP – BG - titanium customized HA - | N.A | 32 | 0 | -0.28 | 0.11 | -0.47 | 0.14 | NA | NA | -0.70 | 0.16 | NA | NA | NA | NA | NA | NA |
| \| *Negative values indicate recession or dimensional reduction.*  *IIP: Immediate Implant Placement; BG: Bone Graft; HA: Healing Abutment; IP: Immediate Provisional; NA: Not Applicable; RCT : Randomized Clinical Trial; BL : Bone Level ; IC : Internal Connection ; EC : External Connection ; PES : Pink Esthetic Score* \| \| --- \| | | | | | | | | | | | | | | | | | | | | |

Supplemental Table 7 : Buccal horizontal alteration
